# Evaluating Information Technology-enabled Precision Prevention Initiatives in Health and Care

**DOI:** 10.1055/s-0044-1800719

**Published:** 2025-04-08

**Authors:** Kathrin Cresswell, Michael Rigby, Stephanie Medlock, Mirela Prgomet, Elske Ammenwerth

**Affiliations:** 1 The University of Edinburgh, Usher Institute, Edinburgh, United Kingdom; 2 Keele University, School of Social, Political and Global Studies and School of Primary, Community and Social Care, Keele, United Kingdom; 3 Amsterdam UMC location University of Amsterdam, Department of Medical Informatics, Meibergdreef 9, Amsterdam, Netherlands; 4 Amsterdam Public Health research institute, Digital Health and Quality of Care Amsterdam, The Netherlands; 5 Australian Institute of Health Innovation, Faculty of Medicine, Health and Human Sciences, Macquarie University, Australia; 6 UMIT TIROL, Private University for Health Sciences, Medical Informatics and Technology, Institute of Medical Informatics, Hall in Tirol, Austria

**Keywords:** Medical informatics, prevention, precision, evaluation

## Abstract

Information technology-enabled precision prevention is a relatively new approach designed to improve population health. It forms an organic development linking principles of optimizing added value from health-related information technology and data systems with clinical aspirations to add longer-term problem prevention to immediate illness treatment. It includes drawing on information technology to identify persons at risk for developing certain conditions and then developing targeted behavioral and psychosocial approaches to modifying the behaviors of individuals or specific groups. We here discuss evaluation challenges associated with information technology-enabled precision prevention approaches to facilitate the development of an empirical evidence base. Challenges associated with measuring the impact of information technology-enabled precision prevention initiatives include considerations surrounding the relevance and fit of external data sources, the accuracy of prediction models, establishing added benefits of preventative activities, measuring pre-post outcomes at individual and population levels, and considerations surrounding cost-benefit analysis. Challenges associated with assessing processes of information technology-enabled precision prevention initiatives include the quality of data used to create underlying data models, exploring processes not necessarily related to each other, evolving social and environmental determinants of health and individual circumstances, the evolving nature of needs and interventions over time, and ethical considerations. If these challenges are attended to in evaluation activities, this will help to ensure that information technology-enabled approaches to precision prevention will have a positive impact on individual and population health.

## 1. Introduction

Health systems are most frequently set up for treatment delivery rather than prevention of disease, despite an increasing recognition that illness prevention is central to tackling the significant challenges facing population health [ 1 , 2 ]. Precision medicine is a relatively new concept and turns the traditional health treatment paradigm on its head - seeking to prioritize prevention of illness by strategically targeting those at risk, or adding prevention of further morbidity to treatment of presenting conditions. As such, precision medicine approaches are beginning to redefine healthcare delivery, by personalizing treatment for individual physiological characteristics, likely drug- and dose-responses, and other personal factors [ 3 ]. Digital health data systems, data-driven decision, and focused presentation of data on external health risks are crucial aspects of precision medicine [ 4 ].

Precision medicine encompasses the interlinkage of two central concepts: precision treatment and precision prevention. Precision treatment focuses on finding the most effective treatment for individuals or specific groups, while precision prevention centers on behavioral and psychosocial approaches to modifying the behaviors of individuals or specific groups [ 5 ], whilst also providing a means of enabling the ‘expert patient’ to optimize personal health lifestyle decisions [ 6 ]. This innovative integrational approach blends traditional bio-physiological data with population and public health data including genomics, biomarkers, lifestyle factors, behavioral patterns, and social and environmental determinants of health [ 7 8 9 10 11 ].

Current developments are largely uncoordinated initiatives to improve health by prioritizing targeted preventive health actions and are dependent on good data and enabling information technology (IT) systems. In practice, they form largely grass roots moves to achieve the vision of Smarter Health and Wellness Models scoped and promoted at policy level by the Organization for Economic Cooperation and Development (OECD) based on energized and interlinked information and communication technologies focused on person-centered action through use of data [ 12 , 13 ].

The progression from early concepts to current precision medicine innovation is linked and enabled by the increasing role of IT and health (and related) informatics. Evidence and rationale are necessary key foundations, and formative as much as summative and outcome evaluation is essential to ensure safety and efficacy. These imperatives form the theme and purpose of this paper.

**Figure 1.**
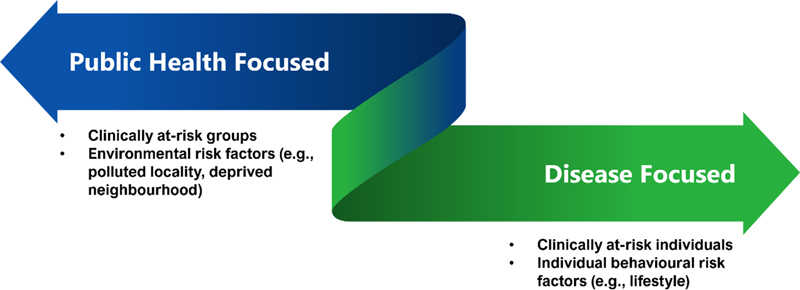
Illustration of the extremes of precision prevention.

We here largely adopt the definition provided by Bíró *et al.* , [ 7 ] defining precision prevention as a *“form of prevention in public health, which includes the activities of personalized prevention and in which health professionals also consider the socioeconomic status or the opportunities offered by psychological and behavioral data of the client when making proposals to maintain or improve the individual's quality of life”* . However, in addition to these public health uses of precision prevention, we also include the use of these methods in therapeutic and primary care to account of holistic disease-focused approaches for individuals ( Figure 1 ).

Precision prevention emphasizes identifying personal predispositions to specific illnesses and conditions, as well as recognizing the impact of social and environmental determinants on health. Examples of approaches may include using data mining or natural language processing of healthcare data to identify individuals at high or low risk and adjusting the level of preventive care accordingly, or tailoring prevention strategies for individuals or groups for whom traditional preventive measures may not be effective. Examples of interventions may include creating customized nutrition plans for those at risk of obesity, designing exercise routines for individuals prone to cardiovascular issues, or implementing preventative mental health measures, like cognitive-behavioral therapy, for those at risk of developing conditions such as bipolar disorder. Precision prevention may also empower individuals to make health protection decisions based on near real time local data, such as avoiding transient localized pollution, ultraviolet risk, or high pollen counts, utilizing clinical guidance on their condition. Such interventions can be digital ( *e.g.* , delivered through an app). At the extreme, precision prevention may be conceptualized as spanning from disease-focused and as public health-focused ( Figure 1 ).

The evaluation of emerging interventions like IT-enabled precision prevention is essential to gauge their impacts and outcomes, including ethics of data linkage, safety and efficacy, economic effects, returns on investment, effects on practitioner and organizational performance, and their influence on patient outcomes. Additionally, assessing the processes and contexts surrounding emerging interventions, such as their development, implementation, and usage, is crucial for refining and advancing precision prevention in public health.

An explorative literature review conducted by the authors showed that the number of studies in PubMed using the term “precision prevention” has been steadily increasing from 400 in 2001 to almost 5,000 in 2023. Most papers describe approaches and examples of precision prevention. Yet, there are very few evaluation studies or reviews surrounding the impact on and processes involved in precision prevention initiatives [ 14 , 15 ]. Underlying reasons may include the emerging nature of the concept and existing challenges in evaluating precision prevention interventions.

Evaluation challenges are indeed complex, involving identifying and capturing an individual's potential ill-health determinants (which, if holistic, need to cover environment and lifestyle as well as physiology and mental wellbeing) and making assessments as to what may have been avoided without precision prevention interventions.

There is currently a lack of evidence around evaluation methods, data coverage, and effectiveness of interventions, leading to uncertainty surrounding decisions of whether initiatives are worth pursuing and what risks need to be mitigated. Having this evidence is, however, the basis for informed decision making and for evidence-based medical informatics [ 16 ].

The aim of this paper is thus to give an overview of existing evaluation challenges associated with emerging IT-enabled precision prevention approaches. We then derive recommendations regarding how to evaluate such initiatives, and highlight research needed to create an empirical evidence base over the next decade.

## 2. Evaluation challenges associated with IT-enabled precision prevention

Approaches to precision prevention can be understood as complex interventions that consist of multiple interconnected elements, including identification of the target group based on classical risk prediction models and genetic susceptibility, and developing targeted strategies to address identified risks [ 17 , 18 ].

**Figure 2.**
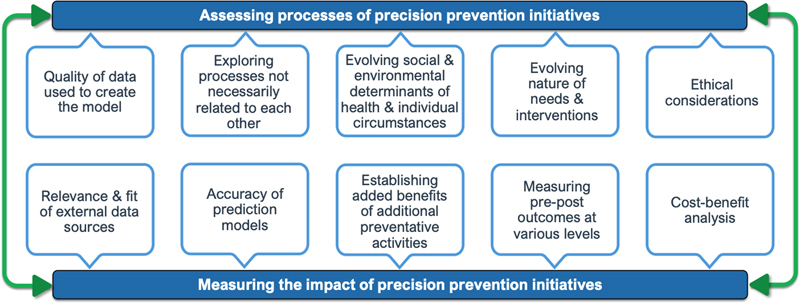
Summary of identified evaluation challenges.

The following paragraphs explore complexities in evaluating impacts and processes of precision prevention interventions ( Figure 2 ).

### 2.1 Challenges associated with measuring the impact of IT-enabled precision prevention initiatives

#### 2.1.1. Relevance, and fit of external data sources

Precision prevention can be enhanced by incorporating external non-medical data sources, including information on environmental pollution and local deprivation indices. If included, it is crucial to evaluate both the reliability and accuracy of these external data sources.

#### 2.1.2. Accuracy of prediction models and establishing added benefits of additional preventative activities

Evaluating how “precise” the precision prevention is should be relatively straightforward; evaluation of the accuracy of prediction models and the value of diagnostic tests is well understood. We know how to evaluate systems that predict risk or stratify according to risk [ 19 , 20 ], but in precision prevention we also need to know how much added benefit the additional preventative activities will have in those specific patients - or, inversely, what the cost is of *not* doing the preventative activities in lower-risk individuals. In other words, it is not enough to know if the risk classification is accurate. Evaluators need to also assess what individuals are doing with that information. For example, genetic testing can identify patients at increased risk of severe neutropenia as a side effect of azathioprine therapy. However, a clinical trial showed less benefit than expected from performing the tests, because clinicians often did not change their prescribing behavior according to the test results [ 21 ].

#### 2.1.3. Measuring pre-post outcomes at various levels

Precision prevention effectiveness considers the effect, safety, and costs of an intervention [ 22 ]. However, randomized controlled trials may not be ethical to use, so pre-post designs may be more suitable [ 23 ]. The effect of a precision prevention intervention can be assessed by measuring outcomes before and after the change ( *e.g.* , in a time series). For example, cancer screening is primarily done at the population level, with a concomitant level of overdiagnosis and potential harms from unnecessary invasive diagnostics and treatment. An alternative would be to predict cancer risk based on genetic and environmental determinants and adjust the intensity of screening according to risk [ 4 ]. This may be particularly effective in cohorts of individuals with predispositions to specific rare cancer [ 25 ]. The effect of this change could be assessed by following a population through the shift from population-based to precision screening.

IT-enabled precision prevention involves a broad spectrum of stakeholders and sectors with varying, at times conflicting, interests [ 26 ]. A critical question arises regarding the level at which to measure the effectiveness of interventions - whether at the individual level, the community level, the societal level, or a combination of these. Precision prevention initiatives are likely to impact on multiple levels, resulting in challenges surrounding what impacts to measure and what processes to explore [ 27 ].

#### 2.1.4. Cost-benefit analysis

Cost-benefit analysis is already challenging in preventative interventions. An intervention such as vaccination may have indirect clinical benefit for people other than the person who was vaccinated, as well as indirect public health benefits such as reallocation of healthcare resources that would have been needed to care for patients who may have otherwise fallen ill [ 28 ]. Some benefits may only occur if enough individuals are vaccinated, such as decline in the rate of new infections or eventual eradication of the disease [ 29 ].

IT-enabled precision prevention adds another layer of complexity: the benefit of tailoring the intervention must justify both the cost of the intervention and the cost of the data processing required to determine who should receive it. If cost savings are realized by *not* performing the preventative intervention for low-risk individuals, a cost-benefit analysis should also consider whether there are indirect benefits that are being lost.

Finally, opportunity costs should be considered. Simple, population-based interventions that benefit many people may have a greater net benefit than a precision intervention that benefits a smaller population [ 10 ]. Evaluation should consider not just the population that receives the intervention, but rather the entire population that is affected by the intervention. This needs to include exploring indirect benefits.

### 2.2. Challenges associated with assessing processes of IT-enabled precision prevention initiatives

New initiatives seeking to innovatively harness the power of IT and computed data systems need formative evaluation to ensure that their intentions will have optimal outcomes and to understand how interventions achieve their effects. Given the inherent nature of precision prevention, whereby interventions are tailored to individuals or specific groups, these issues are likely to vary significantly across contexts. They are also likely to affect the generalizability of findings across various health systems and global regions.

#### 2.2.1. Quality of data used to create the model

IT-enabled precision prevention can be considered as a special case of preventative interventions. The quality of a precision intervention is contingent on the quality of data used to underpin the model used to identify the people or populations who should receive specific preventative care. A first step in developing a precision intervention is the identification of relevant risk factors and biomarkers and ensuring that the most important variables are considered [ 30 ]. Here, it is critical that data are assessed for quality to ensure model validity and robustness [ 31 ]. Model development also needs to consider the effort and cost required to collect the data and routinely evaluate model performance.

#### 2.2.2. Exploring processes not necessarily related to each other

Within IT-enabled precision prevention interventions, there are diverse processes at play, many of which may not necessarily have direct connections with each other. This complexity makes it exceptionally challenging to track and understand how these processes interrelate. Hence, individual interventional elements may be difficult to clearly define, and they may vary across study populations. For example, when considering precision prevention approaches for individuals with pre-diabetes, the target group is likely to vary in relation to phenotype and risk factors, requiring different interventions tailored to individual circumstances [ 32 ]. Therefore, some have argued for the need to develop specifically targeted and contextualized precision prevention interventions for narrowly defined cohorts [ 33 ]. Process assessments must consider and report these specific contexts to facilitate interpretation and transparency of factors leading to success (or otherwise) of interventions.

#### 2.2.3. Evolving social and environmental determinants of health and individual circumstances

Another challenge lies in considering the evolving social and environmental determinants of health and individual circumstances, which are multifaceted and not always straightforward to identify. For instance, when individuals face multiple disease risks simultaneously, the intervention may need to involve parallel strands. To address this, there is an increasing need to draw on social and behavioral sciences to help develop interventions that are tailored to contexts and evolving needs of the target population [ 34 ]. For example, assessing social factors such as loneliness and an individual's ability for self-care becomes pivotal in understanding the datasets and the interplay of factors influencing behavioral change and the provision of formal and informal care [ 35 36 37 38 ].

#### 2.2.4. Evolving nature of needs and interventions

IT-enabled precision prevention interventions are not and indeed should not be static. They need to evolve over time in line with varying needs of different stakeholder groups and emerging evidence. Not only are biological and genetic insights evolving but approaches to data science and computing power are steadily transforming approaches to precision prevention. For example, the notion of risk factors and what should be considered as a risk factor for Alzheimer's disease has been changing over time [ 39 ]. Evaluations need to take this evolution into account, without losing sight of understanding essential elements of interventions.

#### 2.2.5. Ethical considerations

There is also a need to track unintended consequences of IT-enabled precision prevention interventions. For example, efforts may inadvertently perpetuate existing biases and reinforce inequality [ 10 , 40 ]. As a result there is a fine line between tailoring interventions to the needs of specific target groups without leaving other populations behind. Evaluations therefore face a challenge of exploring which groups may have been left out ( *e.g.* , the elderly who may struggle with use of IT-enabled interventions, or those with less information or healthcare access) and where interventional components may need to be adjusted to increase equity.

There is further a lack of consensus regarding what data should be considered as precision prevention. While the pertinent factors will vary depending on the specific context, there is a danger of relying on readily available data rather than the most appropriate data [ 41 ]. This is especially concerning in public health, as it could lead to interventions disproportionately benefiting those who are already well-represented in the data, potentially exacerbating health disparities. For example, in attempts to identify patients with a genetic pattern indicating likely future development of hypertrophic cardiomyopathy, a subset of patients – all African Americans – were later found to be “false positives” [ 42 ]. The data used to identify the at-risk genetic patterns lacked data from African Americans, and therefore failed to account for some normal genetic variants in this subpopulation.

New linkages of different types of data are likely to require concept mapping as a basis for evaluating whether data options are optimal, reliable, and ethical in their interlinkage. For example, ethical issues may arise when collecting data about individuals, ranging from genetics to caring capacity or parallel needs. These have been raised in principle in earlier international studies [ 13 , 36 ]. The overall ethical issues of the design and optimization need assessment, and can draw on existing work in this area [ 43 ].

## 3. Recommendations for future research and practice surrounding IT-enabled precision prevention

Given these challenges surrounding evaluation of impacts and processes in IT-base precision prevention initiatives, there is an urgent need to develop theory-based approaches to inform the real-time and systematic study of interventions. Such efforts will aid drawing on existing experience and help to establish an existing evidence base that can inform strategic development and implementation decisions. This evidence can help to make informed decisions about which programs should take precedence, based on their potential to yield desired outcomes. In doing so, evaluators should build on existing evaluation frameworks of public health and health information technology interventions wherever possible and test their applicability to precision prevention interventions.

Given the complexity of the evaluation task in precision prevention, logic models can be useful to maintain an overview and help to generate hypotheses [ 44 ]. Logic models show relationships among the resources, activities, assumptions and contextual factors, outputs, outcomes, and impact of the intervention [ 45 ]. They can help evaluators understand how precision prevention interventions work in various contexts and what results they may achieve. Here, it is essential to consider a wide spectrum of dimensions, ranging from micro-level individual to macro-level societal contexts.

If these factors are attended to, with existing challenges in mind, appropriate evaluation will help to ensure that IT-enabled precision prevention initiatives achieve maximum positive impact on individual and population health.
